# Supplementary material for: The Cell Membrane of a Novel Rhizobium phaseoli Strain Is the Crucial Target for Aluminium Toxicity and Tolerance
Source: Cells. 2022 Mar 3;11(5):873. doi: 10.3390/cells11050873 (PMC8909678; doi:10.3390/cells11050873)
Supplement: Supplementary file 1 [file cells-11-00873-s001.zip › cells-1605775- sup-final/Table S1.pdf]

Figure S1: Predicted protein topology of (A) AMC88_RS25470 (BON domain-containing protein), (B) AMC88_RS00605 (putrescene-ornithine antiporter protein), and (C) AMC88_RS16850 (DUF1236 domain-containing protein).

Table S1. Details of analysis of GABA and amino acids by LC-MS/MS [HPLC 1260 (Agilent Technologies)-QTRAP6500 (SCIEX)] in positive ionization mode

**compound**	**Q1**	**Q3**	**RT (min)**	**Internal standard**	**IS Q1**	**IS Q3**	**DP**	**CE**
GABA	104.1	87.1	0.5	13C,15N-Ala				
Ala	90.1	44.1	0.5	13C,15N-Ala	94.1	47.1	20	17
Pro	116.1	70.0	0.5	13C,15N-Pro	122.1	75.0	20	19
Val	118.1	72.2	0.5	13C,15N-Val	124.1	77.2	20	13
Thr	120.1	74.2	0.5	13C,15N-Thr	125.1	78.2	20	13
Ile	132.2	86.1	1.1	13C,15N-Ile	139.2	92.1	20	13
Leu	132.2	86.1	1.3	13C,15N-Leu	139.2	92.1	20	13
Met	150.2	104.1	0.7	13C,15N-Met	156.2	109.1	20	13
Phe	166.2	120.2	2.6	13C,15N-Phe	176.2	129.2	20	17
Arg	175.1	70.1	0.4	13C,15N-Arg	185.1	75.1	20	31
Tyr	182.1	136.2	1.4	13C,15N-Tyr	192.1	145.2	20	17
Lys	147.1	84.1	0.4	13C,15N-Lys	155.1	90.1	20	23
