# Supplementary figures and images for: The Cell Membrane of a Novel Rhizobium phaseoli Strain Is the Crucial Target for Aluminium Toxicity and Tolerance
Source: Cells. 2022 Mar 3;11(5):873. doi: 10.3390/cells11050873 (PMC8909678; doi:10.3390/cells11050873)

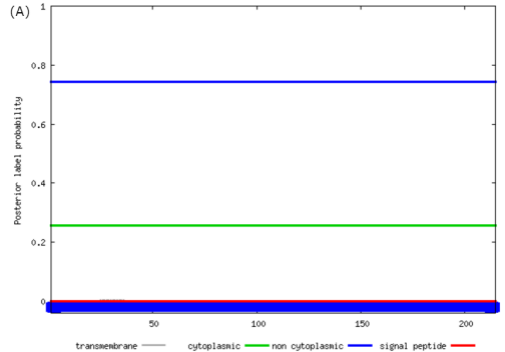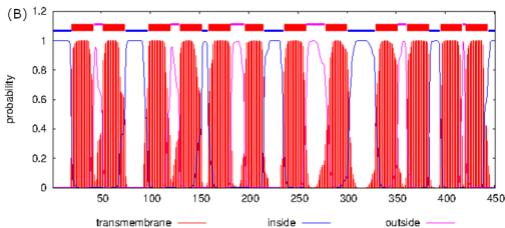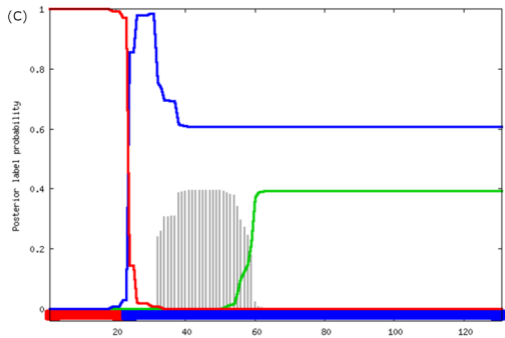

Supplement: Supplementary file 1 [file cells-11-00873-s001.zip › cells-1605775- sup-final/Figure S1.pdf]
